# Peptide Detection of Fungal Functional Amyloids in Infected Tissue

**DOI:** 10.1371/journal.pone.0086067

**Published:** 2014-01-21

**Authors:** Melissa C. Garcia-Sherman, Nataliya Lysak, Alexandra Filonenko, Hazel Richards, Richard E. Sobonya, Stephen A. Klotz, Peter N. Lipke

**Affiliations:** 1 Department of Biology, City University of New York Brooklyn College, Brooklyn, New York, United States of America; 2 Department of Pathology, University of Arizona, Tuscon, Arizona, United States of America; 3 Division of Infectious Diseases, University of Arizona, Tuscon, Arizona, United States of America; New Jersey Medical School, Rutgers University, United States of America

## Abstract

Many fungal cell adhesion proteins form functional amyloid patches on the surface of adhering cells. The *Candida albicans* **A**gglutinin-**l**ike **s**equence (Als) adhesins are exemplars for this phenomenon, and have amyloid forming sequences that are conserved between family members. The Als5p amyloid sequence mediates amyloid fibril formation and is critical for cell adhesion and biofilm formation, and is also present in the related adhesins Als1p and Als3p. We have developed a fluorescent peptide probe containing the conserved Als amyloid-forming sequence. This peptide bound specifically to yeast expressing Als5p, but not to cells lacking the adhesin. The probe bound to both yeast and hyphal forms of *C. albicans*. *Δals1/Δals3* single and double deletion strains exhibited reduced fluorescence, indicating that probe binding required expression of these proteins. Additionally, the Als peptide specifically stained fungal cells in abscesses in autopsy sections. Counterstaining with calcofluor white showed colocalization with the amyloid peptide. In addition, fungi in autopsy sections derived from the gastrointestinal tract showed colocalization of the amyloid-specific dye thioflavin T and the fluorescent peptide. Collectively, our data demonstrate that we can exploit amyloid sequence specificity for detection of functional amyloids *in situ*.

## Introduction

Amyloids are structured insoluble aggregates consisting of many molecules of the same protein. These protein arrays are often associated with disease states such as AL amyloidosis and Alzheimer’s diseases. Nevertheless, there are proteins whose amyloids are a normal part of an organism’s biology [1], [2]. Such functional amyloids exist in many organisms; from prokaryotes to eukaryotes [3]–[6]. They are integral to many biological functions such as cell-cell contacts, biofilm formation, scaffolding and substrate adhesion [6]–[9]. In fact, microbial amyloids are not only important for biofilm formation but also to modulate host-microbe interactions [7], [8], [10]. Many microbes utilize functional amyloids to attach to and colonize the host; which may allow for host infection or commensalism [6], [7], [10].

*Candida albicans* is a fungal member of the human microbiome [11]–[13]. This yeast colonizes the skin, oral, gastrointestinal and urogenital tracts of healthy individuals and can cause nosocomial infections in immunocompromised patients [11], [14], [15]. *Candida* infections can be superficial or systemic, including, respectively oral thrush and often-fatal candidemias [15]. Additionally, *C. albicans* forms mixed fungal-bacterial biofilms with increased resistance to antifungals and antibacterials [16]–[19]. The ability of this fungus to aggregate, adhere to host cells, form biofilms, and even modulate the host response is attributed to amyloid-forming adhesion proteins belonging to the Als adhesin family [7], [20]–[24].

Cell adhesion is critical to both the commensal and infectious states of *C. albicans* [25]–[28]. Among the adhesins, the **A**gglutinin-**l**ike **s**equence (Als) family includes cell wall-linked glycoproteins encoded at 8 loci [29], [30]. Each member of the Als family contain N-terminal Ig-like invasin domains (Ig) which define substrate specificity and a variable number of 36-amino acid tandem repeats (TR domain) that contribute to substrate binding [31]–[34]. There is a glycosylated serine-threonine–rich domain and a GPI anchor that covalently links the protein to the cell [35]. Located between the Ig and TR domains is the T domain, which contains the amyloid-forming sequence [36], [37]. This sequence mediates aggregation of Als proteins into cell surface clusters termed nanodomains composed of functional amyloids [38], [39].

Functional amyloids formed by the *C. albicans* adhesin Als5p have been extensively studied using a *Saccharomyces cerevisiae* expression system [7], [23], [36]–[40]. Soluble Als5p forms amyloid fibers that show characteristic binding of the amyloid binding dyes thioflavin-T, thioflavin-S and Congo red [36], [37]. Amyloid-like patches formed on the cell surface are critical for the functional properties of Als5p, including cell aggregation, biofilm formation, and host immunomodulation [7], [38], [39]. Data from these studies are proving to be a predictive model for studying functional amyloids formed on the surface of *C. albicans in situ* [38], [39], [41]. Recently, amyloids have been demonstrated on fungi in abscesses in the human intestinal tract. Moreover these amyloids bind to human serum amyloid P component, a soluble pattern recognition receptor with anti-inflammatory properties [42]. Thus assessing the role of these amyloids in infections is important for our understanding of fungal disease.

We have recently demonstrated the presence of surface amyloids on fungi in invasive disease in humans, but the nature of these amyloids is unknown. They might be of host or fungal origin, or both, and their composition is unknown. Therefore, we have established a method to label an amyloid sequence and have demonstrated amyloid composed of Als proteins in infected tissue.

## Results

### A fluorescent labeled peptide containing the amyloid sequence from Als5p binds to Als5p-expressing yeast cells

**Figure 1:**
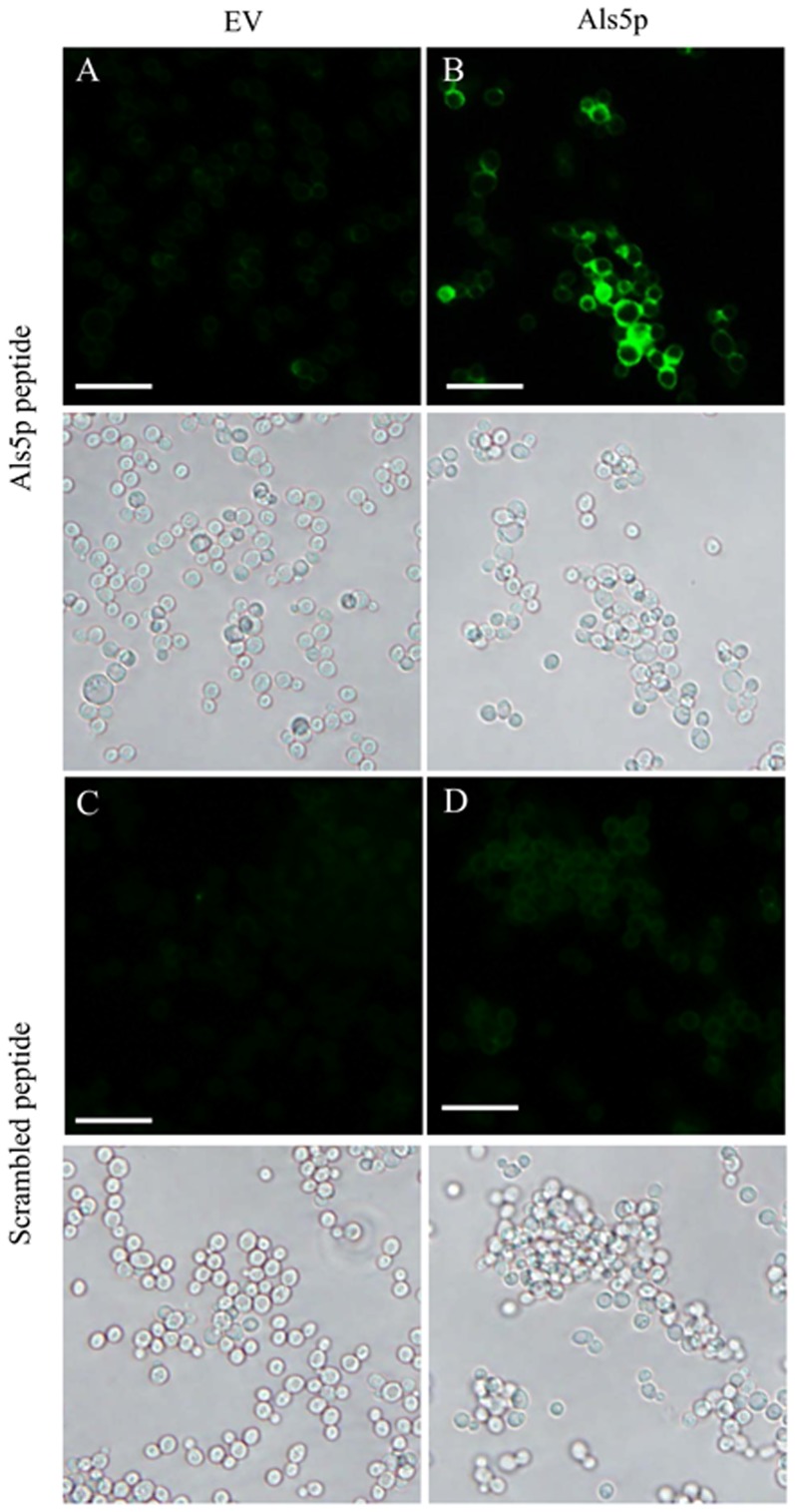
Als5p expressing S. cerevisiae cells stained with fluorescent peptides. *S. cerevisiae* harboring an empty vector (A,C) or expressing Als5p (B, D) were stained with amyloid peptide (200 µg/ml; A, B) or a scrambled-sequence peptide of identical composition (200 µg/ml; C, D). Lower micrographs are bright field images. All scale bars are 30 µm.

Previously we have shown that a peptide containing the amyloid sequence common to Als1p, Als3p, and Als5p (SNGIVIVATTRTV) can form amyloids, and that exogenous peptide can reverse the loss of aggregation in *S. cerevisiae* expressing an amyloid deficient mutation Als5p^V326N^ [36], [39]. We proposed that this reestablishment of aggregation occurs by peptide binding to the mutant protein and serving as a template for amyloid formation [36], [40], [43]. To determine the ability of this protein sequence to bind *S. cerevisiae*-expressing Als5p, we labeled a peptide containing the amyloid forming sequence of Als5p with fluorescein. The labeled amyloid-forming peptide bound specifically to a strain of *S. cerevisiae* expressing Als5p and not to cells harboring the empty vector (Figure 1A and 1B). A scrambled peptide (VITGNTNIRTSVA), containing the same amino acid composition, stained cells poorly (Figure 1C and 1D).

### The Als5p amyloid-forming peptide binds to *C. albicans* in both yeast and hyphal forms

**Figure 2:**
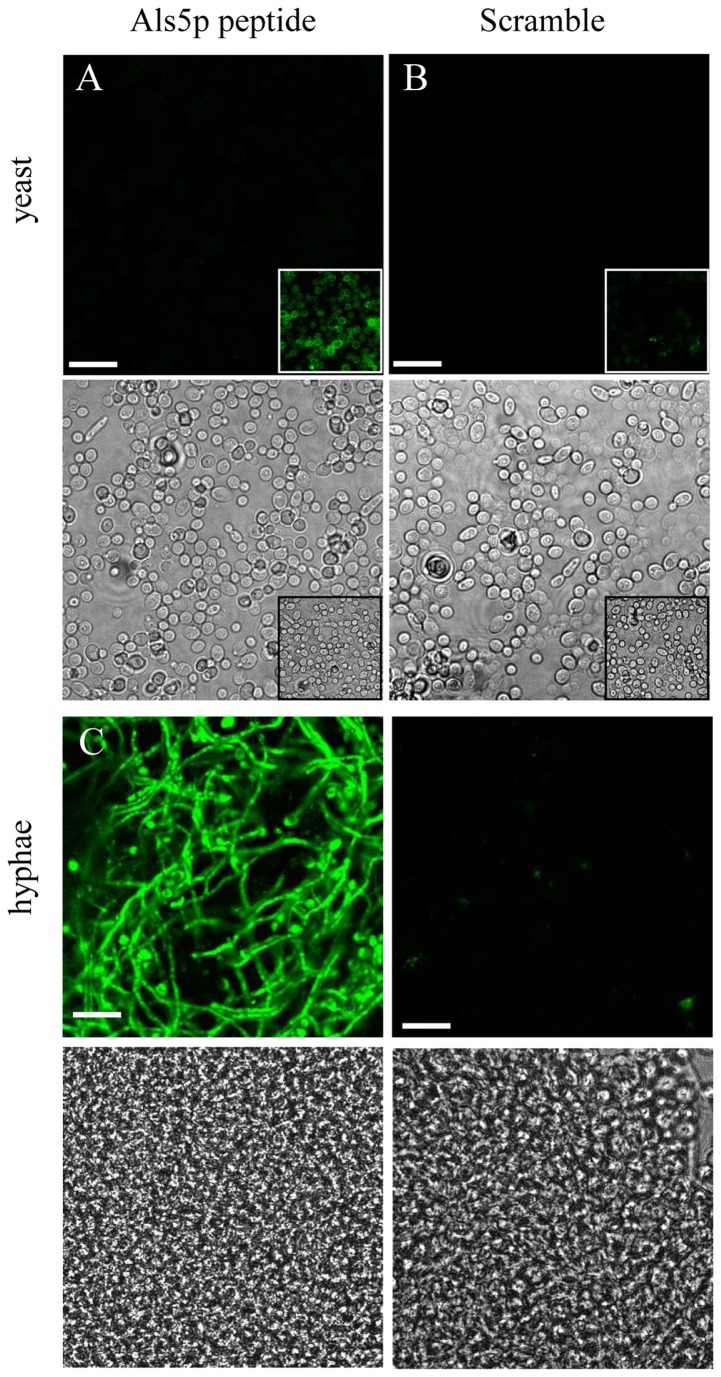
Confocal micrographs of *C. albicans* yeast and hyphae binding Als5p peptide. *C. albicans* cultured in spider medium, with 20 µg/ml (A, C) amyloid or (B, D) scrambled sequence peptide. Fields were imaged containing (A, B) yeast or (C, D) hyphae at a gain of 6.5. Inset images in A and B were acquired at a higher gain of 7.0.

Als1p and Als5p are constitutively expressed at low levels in both yeast and hyphal forms of *C. albicans* [25]. However, the hyphal form of the fungus expresses high levels of Als3p, and in some conditions Als1p as well [21], [44]. Since Als1p, Als3p, and Als5p contain identical amyloid forming sequences, we hypothesized that the Als5p peptide would therefore bind to both hyphae and yeast forms [36]. Fluorescence microscopy revealed a higher level of fluorescence in hyphae versus yeast (Figure 2A and B). This binding was sequence-specific in both cases, because much less cellular fluorescence was seen when the cells were treated under similar conditions with the scrambled peptide (Figure 2B).

### *C. albicans als1/als1 als3/als3* deletion strains show reduced binding of the Als amyloid-forming peptide

**Figure 3:**
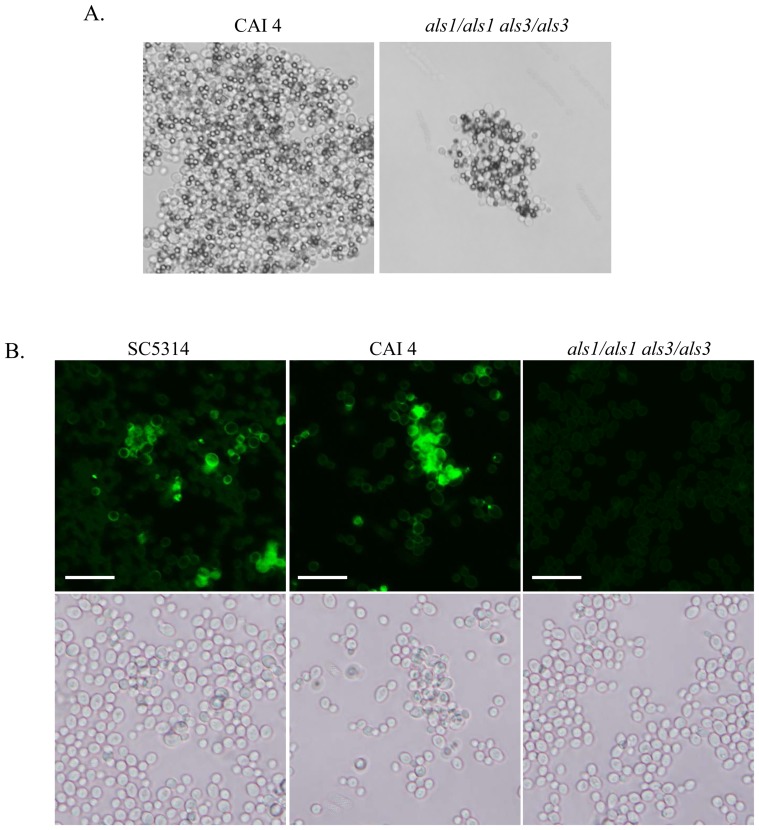
Aggregation and amyloid peptide staining of *C. albicans*. A) Aggregates of wild type (CAI4) and double deletion strains binding with heat denatured BSA-coated magnetic beads. B) The yeast form of *C. albicans* strains, SC5314, CAI4 and *als1/als1 als3/als3* double deletion were probed with 20 µg/ml amyloid peptide. All scale bars are 20 µm.

Als1p and Als3p are major adhesins displayed on yeast phase and hyphae, respectively [21], [44]; therefore, we hypothesized that the amyloid-forming peptide binds not only to the amyloid region of Als5p but also to the homologous regions of these other adhesins. We tested this hypothesis with an *als1/als1 als3/als3* deletion strain. To confirm that the *als1/als1 als3/als3* cells display decreased adhesins we tested for an aggregation defect. The deletion strain aggregated, although less strongly than the parental strain CAI4 (Figure 3A). The labeled peptide stained yeast forms of *C. albicans* CAI4 and SC5314 more brightly than the yeast of the *als1/als1 als3/als3* mutant (Figure 3B).

**Figure 4:**
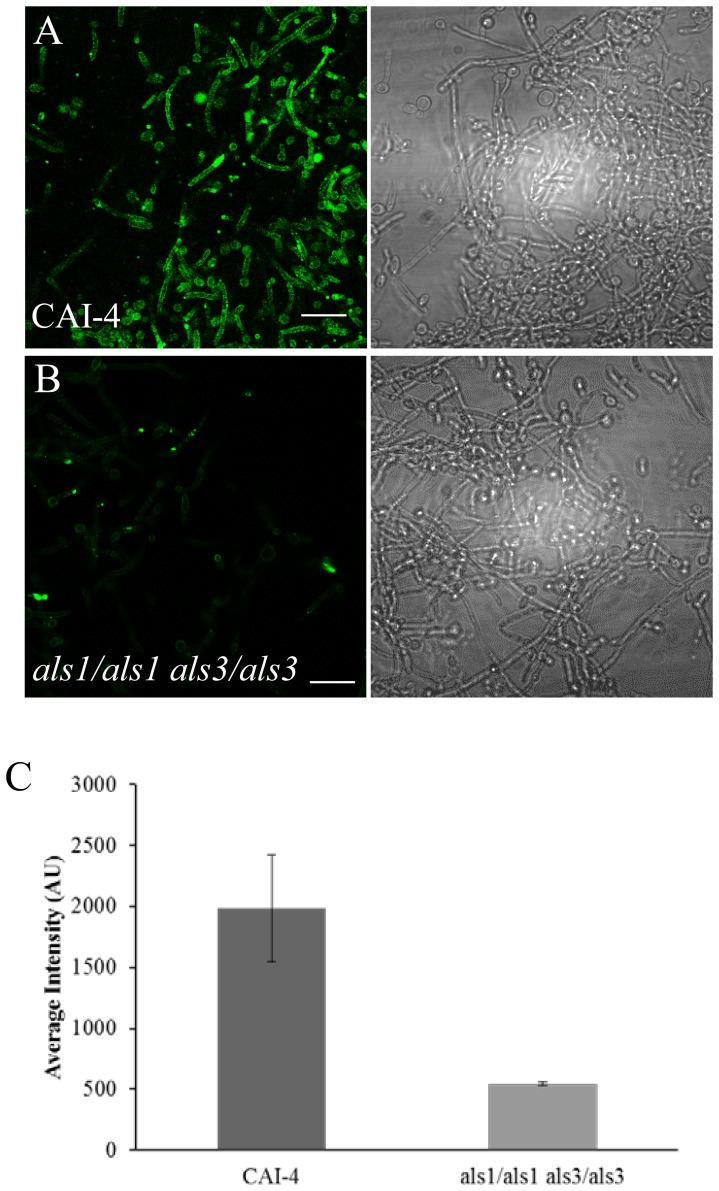
Peptide staining of *als1/als1 als3/als3* deletion strains. All strains were induced to form hyphae by incubating overnight in spider media at 37^°^C. A) CAI4 and B) *als1/als1 als3/als3* double deletion strains were probed with 20 µg/ml amyloid peptide with their corresponding brightfield images (right). All scale bars are 40 µm. C) Quantification of the average intensity of images A and B. Error bars represent standard error of the mean (n = 4; see text for details).

The pattern was also apparent in hyphae induced with spider medium. The hyphal-induced *als1/als1 als3/als3* strain consistently exhibited a 75% decrease in fluorescence when compared to the wild-type CAI4 (Figure 4). Single gene deletion strains of *als1/als1* and *als3/als3* showed variable decreases in labeling (data not shown). Therefore, the majority of the labeling was due to the peptide binding to Als1p or Als3p or both.

### Fluorescent peptide binds specifically to fungi in autopsy tissue from candidiasis patients

**Figure 5:**
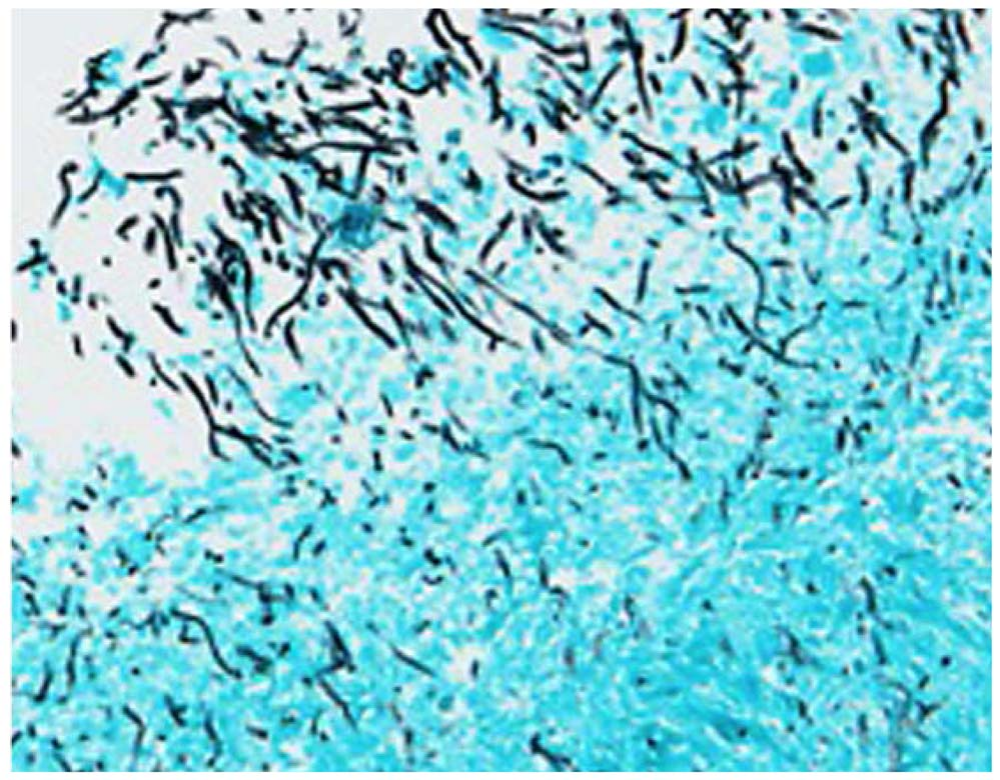
Gomori’s Methenamine Silver stained autopsy section . *Candida* yeast and filamentous forms (hyphae and pseudohyphae) invading human gastrointestinal epithelium at the luminal border [8]. Tissue is a green-blue and the fungi are black.

**Figure 6:**
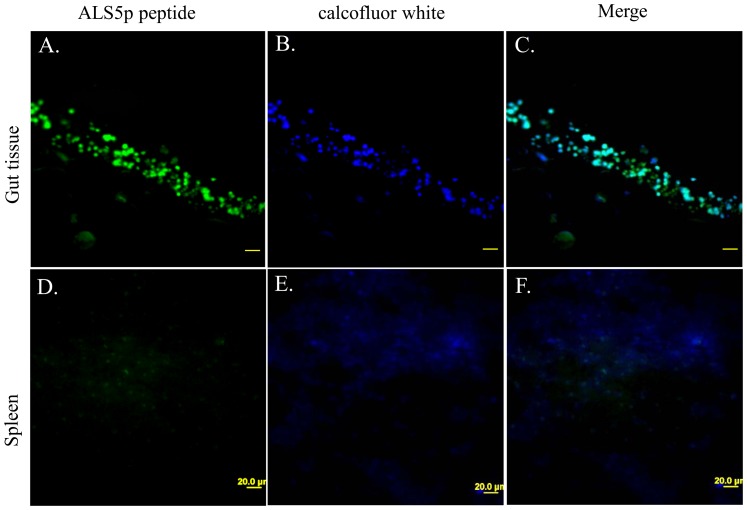
Autopsy sections stained with Als5p amyloid peptide and calcofluor white. A-C) Confocal microscopy of gut section stained with A) amyloid peptide (200 µg/ml), B) calcofluor white, C) merged image. D-F) Spleen section stained with D) amyloid peptide (200 µg/ml), E) calcofluor white, F) merged image.

Amyloid binding dyes bind to yeast in autopsy sections of patients with candidiasis, but the composition of the amyloid is not known [8]. We therefore stained with the Als-specific probe, looking for co-localization with the amyloid binding dyes *in situ*. We stained autopsy gastrointestinal tissue positive for both yeast and filamentous forms of *C. albicans* (Figure 5). Additionally, we stained autopsy spleen samples with the fungal specific dye calcofluor white and with the amyloid peptide. The amyloid peptide efficiently stained yeast in gastrointestinal autopsy sections, but not in an uninfected spleen (Figure 6A and D). In the stained regions, the amyloid peptide co-localized with calcofluor white (Figure 6C). Negative control spleen samples did not show amyloid peptide staining and only diffuse faint calcofluor white fluorescence (Figure 6D-F).

**Figure 7:**
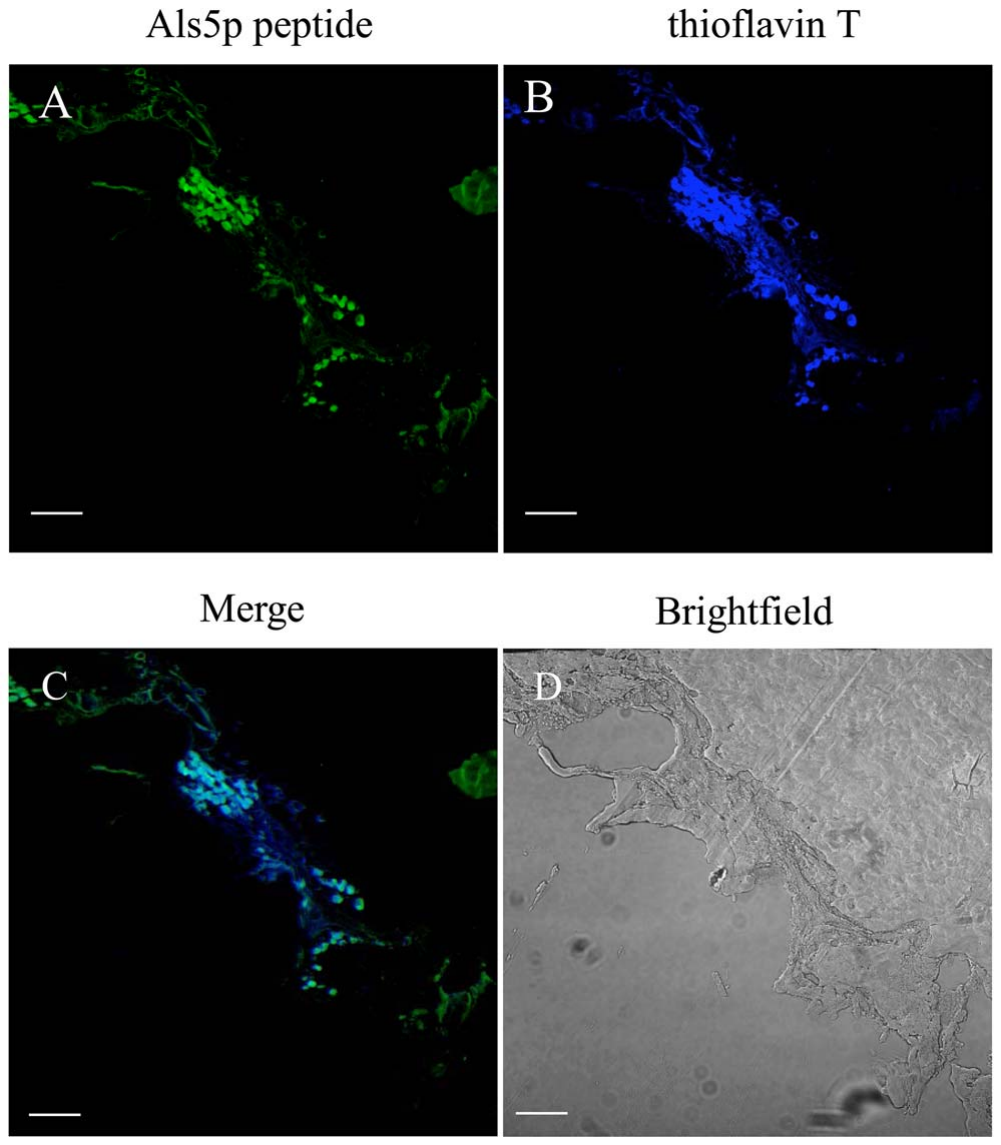
Autopsy section stained with Als5p amyloid peptide and thioflavin T. Confocal images of A-C) gut section stained with A) amyloid peptide (200 µg/ml), B) thioflavin T (100 nM), C) merged image, D) brightfield. All scale bars are 40 µm.

To demonstrate that the peptide was staining the same structures previously observed with amyloid dyes [8], we co-stained autopsy samples from candidiasis patients with thioflavin T and the Als5p amyloid peptide (Figure 7). Yeast and hyphae were positive for both thioflavin T and the amyloid peptide and showed co-localization of the dyes (Figure 7A-C). These results demonstrated that the sequence-specific peptide stained Als amyloids in the tissue.

## Discussion

We have demonstrated that a fluorescent peptide, designed to target the amyloid-forming sequence from the *C. albicans* adhesin Als5p, bound specifically to *S. cerevisiae* cells that express Als5p. This peptide also labeled *C. albicans* yeast and hyphal forms. The peptide showed significantly reduced binding to a strain deleted for the adhesins Als1p and Als3p. Thus, the amyloid peptide bound specifically to proteins that have the same amyloid sequence as Als5p. The peptide co-localized with the fungal-specific dye calcofluor white and with a specific amyloid dye, thioflavin T. These results demonstrate the peptide’s specificity *in situ*. This peptide is a sensitive probe for fungi in autopsy specimens from patients inflicted with candidiasis. We propose that the approach presented here has potential as a new tool for detection of functional amyloids, and specific peptide designs for disease treatment.

We propose that the specific peptide binding observed exploits the sequence specificity of amyloid formation. Both amyloid nucleation and growth are dependent on sequence similarity [39], [41], [43], [45]. Among *C. albicans* Als proteins, Als1p, Als3p, and Als5p share amyloid sequences and thus are predicted to bind the same amyloid peptide [36]. We have previously shown that the amyloid peptide can potentiate *in vitro* amyloid formation, cell aggregation, and biofilm formation [36], [39]. In contrast, a mutated non-amyloid version of the peptide inhibits these activities; therefore, we expected the specific binding of the labeled amyloid-forming peptide. The specificity was supported by several approaches. First only Als-expressing *S. cerevisiae* bound the peptide, and not a scrambled sequence peptide. Second, the peptide labeled *C. albicans* in the pattern expected for expression of Als1p, Als3p and Als5p, showing stronger binding to hyphae than to yeast cells *in vitro* (Figure 2). Collectively, these data indicate that the amyloid sequence-specific peptides can be exploited to design probes to study amyloid forming proteins. This specificity is based on the peptide’s ability to template amyloid formation and to be incorporated in the amyloid structure.

Amyloid dyes, such as thioflavin T and Congo red have been used as pathologists’ gold standard for the detection of amyloids [46], [47]. Although these dyes are inhibitory at high concentrations they can be titrated down to concentrations useful for detection without functional interference [37], [39]. These dyes recognize not only fungal cell wall amyloids, but they also recognize host amyloids. Therefore these dyes would not distinguish the contribution of amyloid from the host or the microbe. The specificity of the Als peptide allows for the resolution of the amyloid source.

Our peptide-based approach allows detection of amyloid without interfering with amyloid function. For instance, this ability could be useful in amyloid-targeted antifungal drug discovery through the ability to mark and quantify amyloid interactions. A similar method has been developed for detecting and quantifying amyloid-beta peptide aggregation by labeling peptide with a quantum nanodot [48]. Presumably a nanodot-coupled Als peptide in a similar assay would lead to discovery of amyloid-targeted antifungal drugs; the signal would decrease as amyloids were inhibited. Thus we could measure the disruption of fungal amyloids in a library screen; thereby indicating to what extent candidate drugs disrupt functional amyloids.

In addition to *in vitro* assays, our peptide approach coupled with nanodot technology and multiphoton microscopy could be utilized as a real-time detection of the initiation and progression of infection by following amyloid kinetics in animal models of infection. This approach has been utilized to track amyloid initiation and maturation in Alzheimer’s disease mouse models [49].

Thus we have used a novel tool to document the presence of a specific fungal amyloid in infections. This new method confirms and extends our previous report of amyloids in autopsy samples from patients with invasive candidiasis [8]. Specifically, functional Als amyloids are present *in situ* in infected tissue. We note that the probe successfully labeled an uncharacterized *C. albicans* strain in these tissues, indicating that this clinical strain contains the highly conserved amyloid forming sequence observed in characterized laboratory strains [36]. In addition, *C. albicans* amyloids bind to serum amyloid P component, a possible modulator of innate immunity, both *in vitro* and *in situ* [8], [42]. Thus the results also support mechanistic studies demonstrating that Als protein amyloid formation is necessary for cell adhesion, fungal aggregation, biofilm formation and binding of serum amyloid P component [8], [37], [39], [40]. The amyloid labeling approach described here may also have more general application for other microbe-derived amyloids [1]–[3], [6].

## Materials and Methods

### Ethics statement

The tissue used in this study was part of a University of Arizona IRB-approved study where we were granted a waiver/exemption for autopsy material. The University of Arizona permission for autopsy is signed by the next of kin and grants use of tissue for research (unspecified).

### Strains and growth conditions

*Candida albicans* strains CAI4 (CAI4-URA3) and *als1/als1 als3/als3* (CJN1348) strains were from Aaron Mitchell (Carnegie Mellon) with the exception of SC5314 which was obtained from ATCC (www.atcc.org) [50]. Als5p (pJL1), Als5p^V326N^ (pJL1^V326N^) and empty vector (pJL1^EV^) *S. cerevisiae* strains were generated as previously described [39].

*S. cerevisiae* strains were cultured as previously described [39]. Briefly, *S. cerevisiae* strains were grown at 30°C on CSM-trp plates enriched with 40 mg/L of adenine and 2% galactose. Liquid cultures were grown at 24°C in galactose and adenine enriched CSM-trp with shaking at 170 rpm to an approximate OD_600_ = 1.0.

*C. albicans* strains were grown at 30°C on YPD agar plates. For induction of hyphae, *C. albicans* cells from overnight cultures were washed 3 times with PBS (137 mM NaCl, 2.7 mM KCl, 10 mM Na_2_HPO_4_, and 1.8 mM KH_2_PO_4_) buffer, and diluted to OD_600_ = 0.5 in Spider medium and incubated for 16 h at 37°C with shaking at 170 rpm [51].

### Peptide labeling

Previously described peptides containing the Als5p amyloid sequence and scrambled sequence were synthesized at The Rockefeller University Proteomics Resource Center (New York, New York) [36], [39]. Peptides were labeled, utilizing fluorescein isothiocyanate adsorbed onto celite, according to the manufacturer’s suggested protocol with minor changes (Sigma-Aldrich, St. Louis, MO). More specifically, the lyophilized peptides were reconstituted in 0.1 M carbonate-bicarbonate buffer, pH 9.0 at a concentration of 1 mg/ml and FITC-celite was added to a final concentration of 1.5 mg/ml and incubated at 24°C for 18h. Celite was removed by centrifugation at 2000 rpm for 3 min. Peptides were aliquoted in tubes at a stock concentration of 1 mg/ml and stored at –80°C.

### Fungal-peptide hybridization

Yeast cultures were grown as described above. 2×10^7^ yeast cells were washed 3 times with PBS and incubated with 200 or 20 µg/ml of peptide in PBS for 20 min. at room temperature. After incubation cells were washed 3 times with PBS. Hyphal-induced cultures were washed 3 times with PBS and incubated at room temperature with FITC-labeled Als5p amyloid peptide (200 µg/ml or 20 µg/ml) for 30 min. Cultures were washed 3 times with PBS and assessed by microscopy.

### Human Tissue-peptide hybridization

Autopsy specimens from patients with histological evidence of invasive candidiasis of the gastrointestinal tract were provided by authors RES and SAK (University of Arizona, Tucson, AZ) in accordance with IRB-approved procedures. Tissues were prepared for staining as previously described [8]. Fixed slides were incubated with 200 µg/ml of peptide for 20 minutes at room temperature. The slides were washed 3X with PBS and stained with 100 nM thioflavin T (in PBS) for 30 minutes. Calcofluor white staining was performed in accordance with the manufacturer’s instructions (Sigma-Aldrich).

### Microscopy and quantification of images

Confocal images were acquired using a Nikon Eclipse 90i confocal microscope with 488-nm excitation and 515-nm emission filters for samples probed with the FITC conjugated peptide. Images of samples stained with Thioflavin T or calcofluor white were acquired with 408-nM excitation and 450-nM emission filter settings. Quantified images were analyzed using the Nikon EZ-C1 Gold version 3.90 software. Fluorescent images, representing a single field per sample, were opened and set to view in 1D graph mode with the x-axis set. The highest fluorescent peak expressed in arbitrary units was noted for four y-axis positions in each field. The mean and standard deviation were determined for the four y-axis positions in four fields.
